# Use of Pharmacogenetic Drugs by the Dutch Population

**DOI:** 10.3389/fgene.2019.00567

**Published:** 2019-07-02

**Authors:** Mohammad A. Alshabeeb, Vera H. M. Deneer, Amjad Khan, Folkert W. Asselbergs

**Affiliations:** ^1^Medical Genomics Research Department, King Abdullah International Medical Research Center, Riyadh, Saudi Arabia; ^2^King Saud Bin Abdulaziz University for Health Sciences, Riyadh, Saudi Arabia; ^3^Division Heart and Lungs, Department of Cardiology, University Medical Center Utrecht, Utrecht University, Utrecht, Netherlands; ^4^Department of Clinical Pharmacy, University Medical Center Utrecht, Utrecht, Netherlands; ^5^Faculty of Population Health Sciences, Institute of Cardiovascular Science, University College London, London, United Kingdom; ^6^Health Data Research UK and Institute of Health Informatics, University College London, London, United Kingdom

**Keywords:** pharmacogenetics, preemptive genetic testing, ADRs, CYP2D6, SLCO1B1, CYP2C19

## Abstract

**Introduction:**

The Dutch Pharmacogenetics Working Group (DPWG) indicated a list of actionable genotypes that affect patients’ response to more 50 drugs; these drugs which show variable effects based on patients’ genetic traits were named as pharmacogenetics (PGX) drugs. Preemptive genetic testing before using these drugs may protect certain patients from serious adverse reactions and could help in avoiding treatment failures. The objectives of this study include identifying the rate of PGX drug usage among Dutch population, estimating the level of users who carry the actionable genotypes and determining the main genes involved in drug’s effect variability.

**Methods:**

Usage of PGX drugs over 2011–2017 by the insured population (an average of 11.4 million) in outpatient clinics in Netherlands was obtained from the publically available GIP databank. The data of 45 drugs were analyzed and their interactions with selected pharmacogenes were estimated. Frequency of actionable genotypes of 249 Dutch parents was obtained from the public database: Genome of Netherlands (GoNL), to identify the pattern of genetic characteristics of Dutch population.

**Results:**

Over a 7 year period, 51.3 million exposures of patients to PGX drugs were reported with an average of 5.3 exposures per each drug user. One quarterof the exposures (12.4 million) are predicted to be experienced by individuals with actionable genotypes (risky exposures). Up to 60% of the risky exposures (around 7.5 million) were related to drugs metabolized by CYP2D6. SLCO1B1, and CYP2C19 were also identified among the top genes affecting response of drugs users (involved in about 22 and 12.4% of the risky exposures, respectively). Cardiovascular medications were the top prescribed PGX drug class (43%), followed by gastroenterology (29%) and psychiatry/neurology medications (15%). Women use more PGX drugs than men (55.8 vs. 44.2%, respectively) with the majority (84%) of users in both sexes are above 45 years.

**Conclusion:**

PGX drugs are commonly used in Netherlands. Preemptive panel testing for CYP2D6, SLCO1B1, and CYP2C19 only could be useful to predict 95% of vulnerable patients’ exposures to PGX drugs. Future studies to assess the economic impact of preemptive panel testing on patients of older age are suggested.

## Introduction

Variability in patients’ response to drug treatment represents a major challenge to medical researchers, clinical practitioners and drug regulatory agencies (Ingelman-Sundberg et al., 2018). The variability may occur as a result of a carriage of different genetic traits that interact with the administered drugs; this interaction may lead to significant drug toxicities and/or lack of drug efficacy (Roses, 2000). Genotypes with potential interactions with certain drugs that necessitate therapeutic intervention or modification such as increasing or decreasing drug dose, stopping the drug or using an alternative are titled as actionable genotypes (Bain et al., 2018). These medications which show variable effects based on patients’ genetic traits can be described as pharmacogenetic (PGX) drugs (Carpenter et al., 2016). Several PGX tests are currently available which allow clinicians to predict a patient’s response to many drug therapies including central nervous system (CNS) agents, cardiovascular, endocrinology agents and many others (Vivot et al., 2015). Action of important therapeutic classes of drugs such as anticoagulants, anti-infective agents, and some pain relievers was confirmed to be affected by multiple interactions with numerous genetic variations. Carriage of such variations may put patients at risk of developing adverse drug reactions (ADRs) (Collins et al., 2016).

The majority of known ADRs, 60% approximately, are triggered via exposure to drugs that are metabolized by enzymes with altered functions induced by genetic mutations (Alagoz et al., 2016). Such types of ADRs which may initiate or extend hospital stays are potentially avoidable. Previous studies have investigated the genotypes frequency of a number of pharmacogenes including CYP2C9, CYP2C19, CYP2D6, SLCO1B1, TPMT, and VKORC1 which showed that more than 90% of individuals carry at least one actionable genotype (Van Driest et al., 2014; Ji et al., 2016). Therefore, implementation of PGX testing has the potential to prevent occurrence of serious drug related events; in some cases leading to death. Prior genetic testing may reduce the episodes of hospitalization as a result of ADR, and minimize the cost of treating avoidable medical conditions (Wong et al., 2010).

The international Clinical Pharmacogenetics Implementation Consortium (CPIC) reported 358 interactions between 127 genes and 226 drugs, and established the needed therapeutic guidelines for 52 drugs up to present^1^. Similarly, the clinical guidelines established by Dutch Pharmacogenetics Working Group (DPWG) identified significant actionable genotypes that affect patients’ response to more than 50 drugs (Samwald et al., 2016). Specific genotyping tests can now be carried out for the reported drugs to protect highly susceptible individuals from serious adverse drug toxicities, to identify low or non-responders and choose appropriate genetic based doses. This is known as personalized medicine where health care providers could tailor medical treatment based on patient’s genetic characteristics (Relling and Klein, 2011; Swen et al., 2011).

Prior knowledge of drug-gene interactions can help in gaining a better understanding of drugs’ mechanism of actions and provide insights for pharmaceutical manufacturer to develop new safer and more effective drugs (Karczewski et al., 2012). Physicians’ compliance with the PGX guidelines and their adherence to the recommendations suggested for each genetic test result is expected to impact treatment effectiveness which may reduce the burden of overall treatment cost. The extent of cost reduction related to ordering PGX tests depends on how common polymorphisms in pharmacogenes exist in certain patient populations, the pattern of diseases and the pattern of drug prescribing in each community (Verbelen et al., 2017).

Despite the determination of important drug-gene interactions, implementation of these findings into clinical practice is still in its early stage; therefore, more efforts are needed to illustrate the benefits of using such PGX tests. Well-designed studies are required to show the potential return of PGX testing prior to drug prescribing and provide detailed therapeutic and cost-effective advantages based on variable demographic data (Cecchin et al., 2017).

This study aimed to determine (1) the major genes involved in drug-gene interactions, (2) the percentage of Dutch population using the drugs listed by DPWG and frequency of genetic variants that affect PGX drugs, in addition to (3) identifying the main prescribed PGX drug category.

## Methodology

The following steps have been taken to estimate actionable genotypes and PGX drug consumption:

(1) Frequency of risk alleles involved in drug interactions among Dutch population was obtained from the public database: (Genome of Netherlands, 2014) (GoNL)^2^. This was used to identify the portion of people at high risk of response alteration when exposed to PGX drugs. Four Dutch biobanks in Amsterdam, Groningen, Leiden, and Rotterdam provided the blood samples for genetic screening. Detailed demographic data of the participants are described by Boomsma et al. (2014). The genetic characteristics available in GoNL database are limited to the Dutch and those with European ancestry only. They represent 85% of the population living in Netherlands (79.3% Dutch and 5.7% Europeans) (Netherlands Population, 2018). 769 Dutch individuals (250 trios) took part in the GoNL project but the genetic dataset shown in this study involved only the genetic profiles of the parents only (*n* = 498; 249 fathers and 249 mothers [average age 63.8 (46–87) and 61.7 (43–86), respectively]. Children data was excluded to avoid the possible confounding effect (bias); since they inherit their genes from their parents. Whole-genome sequencing was performed by BGI Hong Kong. To ensure that GoNL provided accurate estimates of Dutch genotyping data, the results reported in GoNL will be compared to a recent study (Mizzi et al., 2016) that used different genotyping platform (affymetrix DMET^TM^ Plus) and validated its results through using TaqMan SNP Genotyping Assays and/or Sanger sequencing. Mizzi and co-authors investigated selected pharmacogenes among European population, of them 349 were Dutch. GoNL data will also be compared to the Royal Dutch Pharmacists Association (KNMP) genetic database^3^ for variants not reported in Mizzi’s study.
(2) GIP databank^4^, was used to identify the figures of PGX drug usage over the period 2011–2017 by the insured population (an average of 11.4 million) in primary care in Netherlands. Since part of the resident in Netherlands (15%) is not Caucasian (Netherlands Population, 2018), this percentage will be deducted out of the net drug consumption results. Drug consumption data are classified per items issued and per drug users. Repetitive use of a PGX drug by each user was counted as one exposure even though the patient used multiple prescriptions of the same drug.
(3) The consumption data of 45 PGX drugs, described in Table 3, were analyzed and estimations of their interactions with most common genes were made. The PGX drugs listed by DPWG are more than 45 but some of them were excluded due to:
  (a) Restricted to hospital use, e.g., irinotecan (our data focused on outpatient care only).
  (b) Marketed recently (after 2011) in Netherlands, e.g., eliglustat, marketed in 2015.
  (c) Not marketed in Netherlands, e.g., desipramine and warfarin.
(4) Consumption during 2015 and 2017 was further stratified per patient’s sex and different age groups to determine variances in PGX drug consumption between different patients’ categories. The stratified data of other years are not available so we used the 2-year data as an example of the pattern of drug usage rate per different age groups. This has enabled us to pay more attention on the age group of patients with high use of PGX drugs.

Genotypes were translated into predicted phenotypes, e.g., Normal (N), Intermediate (I), Poor (P), or Ultrarapid (U) according to the DPWG guidelines (see text footnote 2).

## Results

Genotyping data of 498 Dutch parents for the common genes, predicted to influence drug response, extracted from the GoNL database are shown in Table 1. The reported results are in line with the Mizzi et al. (2016) findings of 349 Dutch healthy individuals. The screened numbers in both studies may not considered to be representative of the whole population. However, consistency of genetic datasets between both studies provides confidence about the trends of pharmacogenes among the Dutch population. Mizzi et al. (2016) examined only seven genes described in this project. Of which, frequency distribution of 18 SNPs were provided; all showed similar results to GoNL data except CYP2C9^*^3 [Minimum allele frequency (MAF) = 0.20 in Dutch population in Mizzi’s study versus 0.07 in GoNL, *P* = 0.0001]. The average MAF of CYP2C9^*^3 among the tested samples for the other 17 European countries was 0.08 which is consistent to MAF found in GoNL. Also, The frequency of CYP2C9^*^3 shown in KNMP database is in line with the GoNL data, although it is slightly higher (MAF = 0.09 vs. 0.07, respectively).

**TABLE 1:** Frequency of the common variants located in pharmacogenes among Duch population: GoNL data versus other Dutch genetic databases.

**Gene**	**Allele**	**Variant location/Change**	**SNP ID**	**Protein activity**	**MAF (*n* = 498), GoNL**	**MAF in other Dutch sources**	***P*-value**
CYP3A5	^∗^1	6986A > G	rs776746	Increased	**0.06**	0.008	
	^∗^6	14690G > A	rs10264272	Inactive	**0.00**	0.001	
	^∗^7	Deletion	rs41303343	Inactive	**0.00**	0.00	
CYP2B6	^∗^6	516G > T	rs3745274	Decreased or Inactive	**0.25**	0.30	
CYP2C9	^∗^2	430C > T	rs1799853	Decreased	**0.13**	0.11	0.29
	^∗^3	1075A > C	rs1057910	Decreased or Inactive	**0.07**	0.20	0.0001
CYP2C19	^∗^2	19154G > A	rs4244285	Inactive	**0.14**	0.14	0.94
	^∗^3	17948G > A	rs4986893	Inactive	**0.00**	0.00	1.00
	^∗^17	−806C > T	rs12248560	Increased	**0.23**	0.20	0.15
CYP2D6	^∗^2	2850C > T	rs16947	Normal	**0.32**	0.34	0.43
	^∗^4	1846G > A	rs3892097	Inactive	**0.21**	0.20	0.50
	^∗^10	100C > T	rs1065852	Decreased	**0.23**	0.21	0.29
	^∗^17	1023C > T	rs28371706	Decreased	**0.00**	0.00	1.00
	^∗^41	2988G > A	rs28371725	Decreased	**0.09**	0.10	0.74
	^∗^42	3259insGT	rs72549346	Inactive	**0.00**	0.00	1.00
DPYD	^∗^2A	1905+1G > A	rs3918290	Inactive	**0.007**	0.01	0.59
	^∗^13	1679T > G	rs55886062	Inactive	**0.002**	0.00	1.00
	-	1236G > A	rs56038477	Decreased	**0.021**	0.25	
	-	2846 A > T	rs67376798	Decreased	**0.006**	0.007	
FVL	-	1691G > A	rs6025	Decreased	**0.018**	0.03	
HLA	A^∗^31:01	29913298A > T	rs1061235	Idiosyncratic reactions	**0.022**	0.03	
	B^∗^15:02	30699384G > C and 30946148G > A^∗∗^	rs3909184 and rs2844682		**0.006**	0.00	
	B^∗^57:01	31431780T > G	rs2395029		**0.032**	0.034	
	B^∗^5801	31312326T > G and 32257337A > G^∗∗^	rs3134792 and rs4713518		**0.0631**	0.06	
SCLO1B1	^∗^5	521T > C	rs4149056	Decreased	**0.163**	0.14	0.24
TPMT	^∗^2	238G > C	rs1800462	Inactive	**0.001**	0.00	1.00
	^∗^3A (^∗^3B+^∗^3C)	460G > A and 719A > G	rs1800460 and rs1142345	Inactive	**0.021**	0.025	
	^∗^3B	460G > A	rs1800460	Inactive	**0.038**	0.04	0.94
	^∗^3C	719A > G	rs1142345	Inactive	**0.084**	0.07	0.19
VKORC1	^∗^2	−1639G > A	rs9923231	Increased sensitivity	**0.373**	0.37	0.14

*MAF values in red were reported by Mizzi et al. (2016) (*n* = 349), others in blue were obtained from the Dutch KNMP database. SNPs used for HLA typing are tag SNPs. ^∗∗^A haplotype of two SNPs were used to determine genotypes of HLA-B^∗^15:02 and HLA- B^∗^5801.*

**FIGURE 1:**
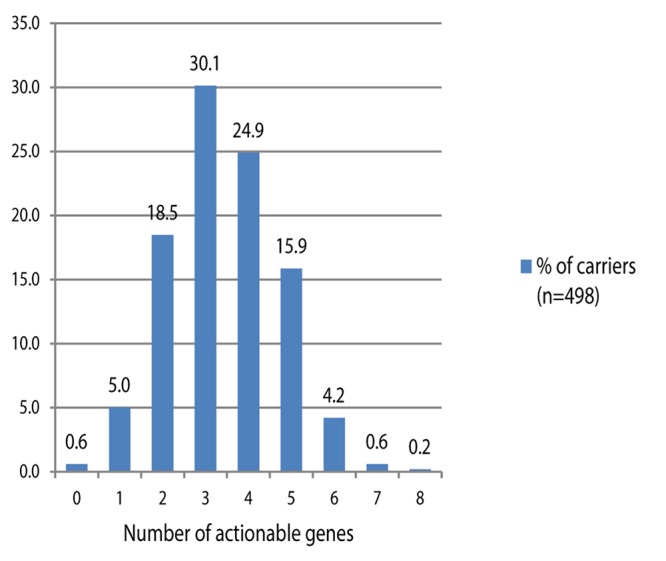
Percentage of Dutch participants carrying 0–8 actionable genotypes.

The levels of genes’ phenotypes were predicted based on the genotypes frequency extracted from GoNL, Table 2. To determine the ultrarapid phenotype of CYP2D6, further knowledge of the copy number of the gene is needed but this was not provided by GoNL; therefore, the frequency of this important phenotype was obtained from KNMP database. MAF of the variants in the pharmacogenes shown in KNMP dataset match the data seen in GoNL. Only 3 out of the tested participants (0.6%) in GoNL were negative for all actionable variants while the vast majority were found carrying at least one or more risky genotypes. Among them, positivity to two, three, four or five genotypes were 18.5, 30, 25, and 16%, Figure 1.

**TABLE 2:** Frequency of pharmacogenes’ predicted phenotypes according to GoNL database (*n* = 498).

**Gene**	**Normal**	**Intermediate**	**Poor**	**Ultrarapid**
CYP3A5	0.002	0.116	0.882	
CYP2B6	0.560	0.386	0.054	
CYP2C9	0.649	0.309	0.042	
CYP2C19	0.675	0.233	0.026	0.066
CYP2D6	0.599	0.259	0.122	0.02
DPYD	0.930	0.050	0.020	
FVL	0.964	0.036	0.000	
HLA-A^*^31:01	0.922	0.076	0.002	
HLA-B^*^15:02	0.994	0.006	0.000	
HLA-B^*^57:01	0.936	0.064	0.000	
HLA-B^*^5801	0.876	0.106	0.018	
SCLO1B1	0.705	0.265	0.030	
TPMT	0.795	0.205	0.000	
VKORC1	0.373	0.506	0.121	

*NB: Classification of the CYPs’ phenotypes based on genetic analysis was made according DPWG guidelines (Swen et al., 2011).*

Over a 7 year period, prescribing of PGX drugs, in total, represented around one quarter (24%) of the total drugs used (384.4 million PGX items issued out of 1,600.2 million prescriptions used), Table 3. Usage of these quantities resulted in 51.3 million exposures of 9.7 million patients [individuals with European ancestry only (85%)] to PGX drugs; an average of 5.3 exposures are estimated per each drug user, Table 4. Of those exposures, one quarter (12.38 million, 24.1%) have clinical significance that may result in reduced drug efficacy or possibly lead to ADRs. Among the screened pharmacogenes interacting with PGX drugs, CYP2D6 was determined as the top gene influencing patients’ response. More than seven million exposures are predicted to involve users with CYP2D6 actionable genotypes which represent 60% of the total risky exposures over 7-year interval. Also, the polymorphisms in SLCO1B1, which codes for statin transporter protein, and CYP2C19, that encode a metabolizing enzyme of multiple drugs, have shown high rates of interaction with used drugs in Netherlands (about 22% and 12.35% of the significant exposures, respectively).

**TABLE 3:** Issuance of PGX drugs over 7-year period (2011–2017) in Netherlands.

**PGX Drugs**	**Issuance (Thousand)**	**%**	**Therapeutic area**
6-Mercaptopurine	136	0.04	oncology
Abacavir	116	0.03	infectious
Acenocoumarol	9,149	2.38	Cardiovascular
Allopurinol	6,210	1.62	other
Aripiprazole	1,625	0.42	psychiatry/neurology
Azathioprine	1,093	0.28	oncology
Capecitabine	297	0.08	oncology
Carbamazepine	3,033	0.79	psychiatry/neurology
Clopidogrel	10,195	2.65	Cardiovascular
Contraceptives with Estrogen	7,842	2.04	endocrinology
Efavirenz	145	0.04	infectious
Flecainide	1,806	0.47	Cardiovascular
Flucloxacillin	2,459	0.64	infectious
Fluorouracil	312	0.08	oncology
Haloperidol	2,678	0.70	psychiatry/neurology
Metoprolol	61,224	15.93	Cardiovascular
Proton Pump Inhibitors (PPI)	110,147	28.66	gastroenterology
Opioids	28,559	7.43	analgesic/anaesthesiology
Phenprocoumon	1,821	0.47	Cardiovascular
Phenytoin	1,028	0.27	psychiatry/neurology
Pimozide	599	0.16	psychiatry/neurology
Propafenone	97	0.03	Cardiovascular
SSRI	27,424	7.14	Psychiatry/neurology
Simvastatin and Atorvastatin	80,653	20.98	Cardiovascular
Tacrolimus	977	0.25	other
Tamoxifen	1,193	0.31	oncology
Tegafur	0.812	0.00	oncology
Thioguanine	53	0.01	oncology
Tricyclic antidepressants	15,034	3.91	psychiatry/neurology
Venlafaxine	7,698	2.00	Psychiatry/neurology
Voriconazole	24	0.01	Infectious
Zuclopenthixol	722	0.19	psychiatry/neurology
Total PGX drugs issued	384,351		
Total prescribed drugs (PGX and non-PGX drugs)	1,600,241		
% of PGX drugs among all medications	24.02%		

**TABLE 4:** Number of patients’ exposed to PGX drugs from 2011 to 2017 and the genes involved.

**Gene**	**PGX Drug (s)**	**Column A: % of patients with actionable genotype / haplotype**	**Column B: Exposures of all patients to PGX drugs**	**Column A x Column B: Estimated exposures in users who carry actionable genotypes (%)**
CYP3A5	Tacrolimus	11.85(N+I)	261,514	30,983⁢(0.25)
CYP2B6	Efavirenz	43.98(I+P)	27154	1,1941⁢(0.10)
CYP2C9	Phenytoin	35.1(I+P)	50,133	17,597⁢(0.14)
CYP2C19	Citalopram, Clopidogrel, Escitalopram, Esomeprazole, Imipramine, Lansoprazole, Omeprazole, Pantoprazole, Sertraline, Voriconazole	9.88(I+P+U)	15,471,291	1,528,438⁢(12.35)
CYP2D6	Amitriptyline, Aripiprazole, Clomipramine, Codeine Doxepin, Flecainide, Haloperidol, Imipramine Metoprolol, Nortriptyline, Oxycodone, Paroxetine Pimozide, Propafenone, Tamoxifen, Tramadol Venlafaxine, Zuclopenthixol	40.1(I+P+U)	18,638,754	7,467,420⁢(60.31)
DPYD	Capecitabine, Fluorouracil, Tegafur	7.00(I+P)	275,923	19,301⁢(0.16)
FVL	Contraceptives with Estrogen	3.6(Het+Hom)	2,561,819	92,226⁢(0.74)
HLA	Carbamazepine	38.54(Het+Hom)	245,493	96,063⁢(0.16)
	Abacavir, Flucloxacillin	6.4(I+P)	1,729,046	110,659⁢(0.89)
	Allopurinol	12.45(I+P)	692,108	86168⁢(0.70)
SCLO1B1	Atorvastatin, Simvastatin	29.52(I+P)	9,226,089	2,723,364⁢(22.00)
TPMT	6-Mercaptopurine, Azathioprine, Thioguanine	20.5(I+P)	193,641	39,696⁢(0.32)
VKORC1	Acenocoumarol, Phenprocoumon	12.05⁢(P)	1931864	232,755⁢(1.88)
Total			51,304,829	12,380,754⁢(100)

*N, Normal metabolism/function; I, Intermediate; P, Poor; U, Ultra rapid; Het, Heterozygous; Hom, Homozygous.*

**FIGURE 2:**
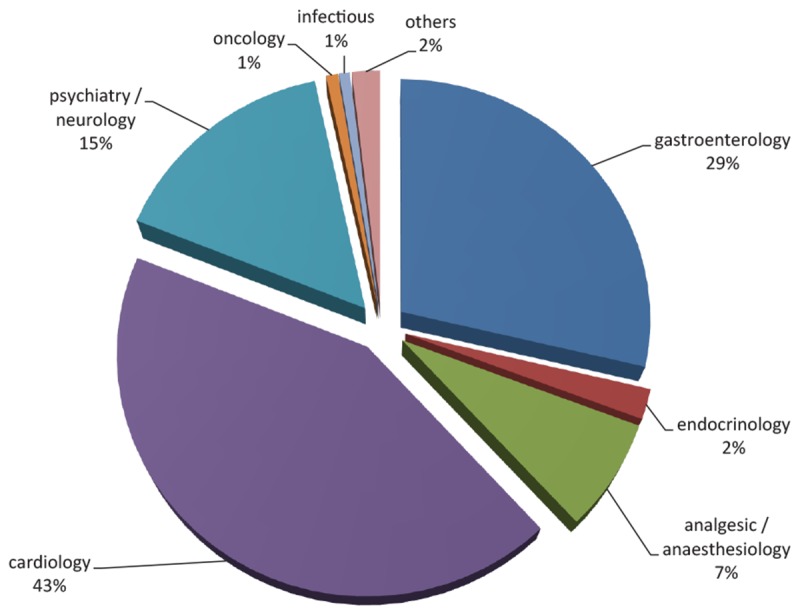
Level of consumptions of different PGX medication classes.

Cardiovascular drugs such as statins, e.g., simvastatin and atorvastatin were the top PGX drug class prescribed (43%), followed by gastroenterology agents, in particular proton pump inhibitors (PPI), e.g., omeprazole and pantoprazole, and psychiatry/neurology medications (29% and 15%, respectively), Figure 2. Use of analgesic/anesthetics medications were rated as fourth class (7%). Metoprolol and simvastatin were the most issued PGX drugs, followed by omeprazole and pantoprazole (16, 15, 14, and 10%, respectively).

Further analysis of combined use in 2015 and 2017 based on different sex showed higher level of PGX drugs usage by women than men (55.8% for women vs. 44.2% for men), Table 5. Intake of these medications was found to be more common in older age groups than younger age groups. The top drug users in both sexes were the group aged between 45 and 64 years (percentage of usage was 36 and 31% for men and women, respectively). In total, more than 84% of PGX drug prescriptions were issued to patients older than 45 years.

**TABLE 5:** Consumption of PGX drugs per sex and specific age groups.

	**Number of PGX drug users per different Age groups**								
	
	**0–4**	**5–14**	**15–24**	**25–44**	**45–64**	**65–74**	**75 +**	**Total**	**% per sex**
**2015**									
MEN	9,916	24,324	69,230	377,305	1,412,311	1,046,979	951,502	3,891,567	44.7
%	0.25	0.63	1.78	9.70	36.29	26.90	24.45		
WOMEN	8,240	36,071	185,101	562,921	1,599,198	1,071,277	1,345,702	4,808,510	55.3
%	0.17	0.75	3.85	11.71	33.26	22.28	27.99		
Total	18,156	60,395	254,331	940,226	3,011,509	2,118,256	2,297,204	8,700,077	
%	0.21	0.69	2.81	10.70	34.77	24.59	26.22		100.0
**2017**									
MEN	11,156	23,894	70,700	366,669	1,418,338	977,954	1,029,667	3,898,379	43.8
%	0.29	0.61	1.81	9.41	36.38	25.09	26.41		
WOMEN	10,245	35,511	442,109	551,128	1,459,159	1,123,780	1,378,342	5,000,276	56.2
%	0.20	0.71	8.84	11.02	29.18	22.47	27.57		
Total	21,401	59,405	512,809	917,798	2,877,498	2,101,735	2,408,009	8,898,655	
%	0.24	0.67	5.76	10.31	32.34	23.62	27.06		100.0
**2015+2017**									
MEN	21,072	48,218	139,930	743,974	2,830,649	2,024,933	1,981,169	7,789,946	44.2
**%**	0.27	0.62	1.80	9.55	36.34	25.99	25.43		
WOMEN	18,485	71,582	627,210	1,114,049	3,058,357	2,195,057	2,724,044	9,808,786	55.8
%	0.19	0.73	6.39	11.36	31.18	22.38	27.77		
Total	39,557	119,800	767,140	1,858,024	5,889,007	4,219,991	4,705,213	17,598,732	
%	0.22	0.68	4.36	10.56	33.46	23.98	26.74		100.0

## Discussion

Usage of PGX drugs may expose patients to increased risk for ADR (Wang, 2010). In addition, treatment failures are expected when drugs are used without prior identification of the exact patients’ genetic characteristics (Blakey and Hall, 2011). Ordering genetic testing to all patients prior to usage of PGX drugs might be the ideal approach to avoid preventable drug toxicities and to improve drug efficacy, therefore, its implementation is the ultimate mean to accelerate practicing precision medicine. Although, preemptive genetic testing is highly recommended (Caudle et al., 2014; Bank et al., 2018b) to predict patient safety, its high cost may prevent health care authorities to propose it as a standard practice in health institutes. The burden on health budget will be huge when genotyping of every patient is requested. Therefore, at this stage, determining the level of consumption of these specific drugs could guide the prioritization process of patients who are of utmost need for genetic testing.

This study is intended to assess the frequency of actionable genotypes among Dutch population and identify the rate of usage of PGX drugs and further identify the frequent users based on their disease type, age group, and sex. The findings of the study were used to set up a recommendation that focuses on certain age groups. A matter of utmost importance, this study shortlisted the main genes of interest which affect the majority of used PGX drugs based on actual large scale drug usage in Netherlands. In contrast to our approach, the previous studies conducted by Van Driest et al. (2014) and Ji et al. (2016) suggested a panel of genes based on their understanding of gene functions and their knowledge of interaction between gene products and the selected PGX drugs. Familiarity with functional consequences of actionable genotypes of different pharmacogenes is needed to identify the most important genes for preemptive testing, however, incorporating this knowledge with the information of PGXs drugs usage rate represents a better method of guided selection of gene panels since it reflects the real need for drug-related genetic testing in each unique population. As a result of different type of diseases and drug prescribing patterns in other countries, such project is suggested among population with different ethnicities to determine the best pharmacogenes for preemptive testing.

Previous surveillance in the United States revealed that 91% of European Americans (EA) and 96% of African Americans (AA) (average age is 64 and 60, respectively) carry genetic variations that could put them at risk of an ADR upon receiving PGX drugs (Van Driest et al., 2014), these risks are predictable and can be prevented when patients being tested for prior to drug administration. In addition, a more recent study that involved 1013 non-Hispanic white United States individuals indicated that 99% of the participants (median age = 56 yeas) inherit at least one actionable variant and 58% of them were found positive for three or more drug affecting variants (Ji et al., 2016). These results were supported by our findings where 99.4% of the genotyped Dutch individuals carried one or more actionable variants. Our data showed that 76% of the participants were positive for three or more risky genotypes. In line with adult findings, pharmacogenes’ screening of 98 pediatric patients revealed that 95% of them had at least one pharmacogenomic variant (Cohn et al., 2017).

Results of this study indicated that exposure of patients to PGX drugs is common in Netherlands with an average of five exposures per patient over 7-year period among users who have actionable genotypes. This finding is similar to the results reported by an American study where half of the participants had one incident experience while one quarter to one third of them had two or more serious PGX exposures over a 4 year period (Samwald et al., 2016). In another study that involved an approximate 53,000 United States patients, 64.8% of them were exposed to one PGX drug within 5 years, whereas 5.9% of the individuals had five exposures (Schildcrout et al., 2012). In the Vanderbilt study which recruited 10,000 patients over 3 years in the so called PREDICT program, 42% of the participants had evidences of exposures to PGX drugs (Van Driest et al., 2014). Although our study covered a longer period (7 years) than PREDICT program, the estimated level of exposure was lower in the Dutch patients (24.1%), but this level is predicted to be raised over time. Each of these exposure episodes to PGXs drugs represents a chance to minimize related ADRs or optimize therapeutic plan.

Selection of the 45 PGX drugs included in this study was guided by the DPWG recommendations. Although CPIC and DPWG share consistent guidelines for the majority of reported interactions between actionable genotypes and PGX drugs, the criteria and methodology for ranking are different (Bank et al., 2018a). Therefore, differences in ranking between both groups may exist in term of level of evidence that support each interaction pairs. For example the claimed interaction between metoprolol with CYP2D6 was classified by DPWG as level 4, this is the highest rank of evidence based on published controlled studies of good quality (Deneer and van Schaik, 2013). In contrast, CPIC classified the evidence of metoprolol-CYP2D6 interaction as level C (A and B levels are the top ranking, (CPIC web page, see text footnote 1). In the case of atorvastatin- SCLO1B1 interaction, limited evidence is available that support an impact of 521T > C variant on atorvastatin-induced myopathy (Pasanen et al., 2007) and therefore was ranked by CPIC as level D, however, DPWG guidelines recommended genetic testing if additional risk factors exist (KNMP genetic database). Patients with high risk involve females, elderly people, those receiving high doses or with lower hepatic or renal function, concomitantly using medications that inhibit CYP3A4 (e.g., clarithromycin) or SLCO1B1 (e.g., gemfibrozil) (Ramsey et al., 2014).

Our study showed high consumption of PGX drugs that belongs to certain therapeutic classes, in particular, cardiovascular agents (metoprolol and statins) and the PPIs. Cardiovascular medications were also found to be the most commonly used therapeutic agents among patients aged more than 65 in the study conducted by Samwald et al. (2016). They also reported PPI group (omeprazole and pantoprazole) as the second drug category used in their study which is similar to our findings. Extra caution need to be taken when prescribing these medications through ordering the appropriate genetic tests and following the recommendations stated by DPWG guidelines. In younger patients, analgesic drugs were used more frequently in both Samwald et al. (2016) and Ji et al. (2016) studies which involved United States population. The latter study found that 75% of participants had an exposure to both tramadol and codeine or at least to one of them.

As identified in this project, CYP2D6, SLCO1B1, and CYP2C19 were the most commonly genes predicted to affect response of Dutch population to the used PGX drugs, hence, genotyping of these nominated genes before drug intake (preemptive testing) may significantly reduce emergence of ADRs and possibly optimize therapeutic outcomes. Similar to our findings, a recent study in the United States reported these three genes proposed in this study in addition to those affecting warfarin metabolism (VKORC1/CYP2C9) to be part of the suggested gene panels to be tested prior to cardiac angiography intervention (Dong et al., 2018). These five genes were exclusively studied by Ji et al. (2016) on 1013 subjects in the United States and emphasized that 79% of the participants carried actionable CYP2D6 genotypes. In contrast, our findings showed that 40% of Dutch population carry one or more CYP2D6 actionable variants. Similarly, the frequency of SLCO1B1^*^5 allele in the Dutch and the United States individuals involved in both studies was exactly the same (30%). Slightly lower frequency (26%) of this allele was noted in the study that involved larger number (10,000) of United States individuals (Van Driest et al., 2014). On the other hand, CYP2C19 actionable genotypes were more prominent in the United States individuals than that seen in Dutch population (28.5 vs. 10%, respectively). Warfarin is not used in Netherlands, however, testing for VKORC1 gene is advisable for both of the warfarin alternatives (acenocoumarol and phenprocoumon) commonly used by Dutch patients. In Netherlands, testing for CYP2C9 is sometimes suggested for patients on phenytoin; in particular, in patients with high phenytoin levels. Genotyping patients for CYP2D6, SLCO1B1, and CYP2C19 only provide the necessary information of possible interactions with 95% (60.31% + 22.00% + 12.35%, respectively) of the prescribed drugs in Netherlands. This information is essential to intervene before exposing patients to unnecessary risk. Knowing the enzymatic level of function based on characterization of gene activity as in the case of CYP2D6 would allow physicians to initiate proper medication and dose at an early stage of drug therapy. For example, CYP2D6 poor metabolizers are advised to use an alternative to amitriptyline (TCAs) or to use half of the recommended dose to avoid risk of adverse reaction which may result due to the elevated drug concentration in blood stream; it could be increased up to 2–3 times the normal level, even though normal doses are used in patients with low gene function (Halling et al., 2008); therefore, selective serotonin reuptake inhibitors (SSRIs), e.g., sertraline and citalopram are suggested to be used instead (Swen et al., 2011). Similarly, patients with poor CYP2D6 gene activity who are using codeine need to be instructed to use alternatives medications such as acetaminophen, non-steroidal anti-inflammatory drugs (NSAID) or morphine to achieve adequate pain relief (VanderVaart et al., 2011). Currently, a number of United States health institutes including universities (in Vanderbilt, Florida and Illinois), medical centers (in Vanderbilt, Mount Sinai and Mayo Clinic) and several hospitals (e.g., St. Jude Children’s Research Hospital and Shands Hospital) started reactive or preemptive testing for certain gene panels (Pulley et al., 2012; Crews et al., 2012; Nutescu et al., 2013; Hoffman et al., 2014; Weitzel et al., 2014; Shuldiner et al., 2014; Dunnenberger et al., 2015); of which the five genes described above are the commonest recommended for testing. Other suggested genes for testing were TPMT, CYP3A5, DPYD, UGT1A1, HLA-B IFNL3, and CYP4F2. The latter two genes were not included in our study as their actionable variants suggested by CPIC were not considered by DPWG guidelines which we adhered to.

Although, our data showed limited rate of usage of antineoplastic agents and chemotherapy (>1%), it is crucial to do genetic testing to avoid extreme toxicities related to some of these drugs, e.g., capecitabine. In addition, genetic profiling may help in tailoring therapies of cancer patients which possibly provide a better chance to treat them successfully (Patel, 2016). Our data indicated that females are using PGX drugs more than males (55.8% vs 44.2%, respectively). Identical results were reported to women (55.7%) in Samwald et al. (2016) the study which analyzed 72 million United States participants.

The highest level of exposure to PGx drug seen in our study was in patients aged 45 or above, who consumed the majority of PGX medications (84%). Of which, the patients aged 45–64 were the top users (33.5%). This finding is consistent with the results reported by Samwald et al. (2016) who emphasized that patients with age group 40–64 had the highest PGX drugs usage rate (37.9%). This may support offering the genetic tests to this targeted group at the meantime to customize health budget until the cost of genetic testing get reduced. However, mandatory tests must continue to be offered for selected younger individuals who may use drugs of serious toxicities, e.g., Abacavir, Capecitabine. The limitation of age breakdown analysis in this study is that the given age groups were divided into 7 groups only (0–4, 5–14, 15–24, 25–44, 45–64, 65–74, and <75) similar to the age distribution available on GIP database. It might be more helpful to further breakdown PGX drug usage over short age periods such as 5 or 10-year periods rather than 20 year age groups as in the case of 25–44 and 45–74 year subgroups.

In conclusion, the level of patients’ exposures to PGXs drugs reported here may encourage Dutch health institutes to offer panel-based preemptive testing with possible wider implementation of DPWG guidelines in the future. Future studies should evaluate cost-effectiveness of preemptive testing in routine clinical care, preferably in selected age groups using limited number of pharmacogenes.

## Author Contributions

MA, FA, and VD worked together in setting the study objectives, designing the project, and analyzing the data. FA suggested the main concept about the project and highlighted its importance. MA took the lead in collecting the data related to drug consumption and identifying the frequency of SNPs in pharmacogenes that characterize Dutch population. VD did a great job in providing the currentDPWGguidelines and deciding the actionable drugs. AK participated in the final approval of the version to be published. The principal author wrote the manuscript which was reviewed and modified by all co-authors.

## Conflict of Interest Statement

The authors declare that the research was conducted in the absence of any commercial or financial relationships that could be construed as a potential conflict of interest.

## Footnotes

1 https://cpicpgx.org/genes-drugs/

2 http://www.nlgenome.nl/

3 https://www.knmp.nl/

4 https://www.gipdatabank.nl/databank.asp
